# Fatores que Afetam a Trombose da Falsa Luz na Dissecção Aórtica Tipo B

**DOI:** 10.36660/abc.20220939

**Published:** 2023-07-20

**Authors:** Qian-hui Tang, Jing Chen, Han Yang, Zhong Qin, Qiu-ning Lin, Xiao Qin

**Affiliations:** 1 Department of Vascular and Endovascular Surgery The First Affiliated Hospital Guangxi Medical University Guangxi China Department of Vascular and Endovascular Surgery – The First Affiliated Hospital of Guangxi Medical University, Guangxi – China

**Keywords:** Dissecção Aórtica, Trombose, Luz

## Abstract

**Fundamento:**

A trombose completa da falsa luz facilita a remodelação da dissecção aórtica tipo B (DATB). As características morfológicas afetam a trombose na falsa luz.

**Objetivos:**

Discutir os fatores pré-admissão presentes, que influenciam a trombose da falsa luz em pacientes com DATB.

**Metodologia:**

Ao todo, 282 pacientes diagnosticados com DATB em nosso hospital foram estudados, no período entre janeiro de 2008 e dezembro de 2017. Os indivíduos foram divididos em um grupo trombótico e um grupo não trombótico, com base na detecção de qualquer trombo na falsa luz. Analisamos as diferenças entre os dois grupos com relação aos dados clínicos, o comprimento vertical da dissecção e o diâmetro da aorta. Valores de p < 0,05 foram considerados estatisticamente diferentes de modo significativo.

**Resultados:**

Diferenças significativas entre o grupo trombótico e o grupo não trombótico foram encontradas com relação à idade (53,92 ± 11,40 vs. 50,36 ± 10,71, p = 0,009) e proporção de pacientes com insuficiência renal (7,83% vs. 16,38%, p = 0,026). Nas zonas 3–9, o diâmetro da luz verdadeira do grupo trombótico foi significativamente maior do que no grupo não trombótico (p < 0,05). A análise de regressão logística binária mostrou que o diâmetro da luz verdadeira na zona 5 e a insuficiência renal foram preditores independentes de trombose da falsa luz.

**Conclusões:**

A idade e a função renal estiveram associadas à trombose na falsa luz. Potencialmente, a diferença entre o diâmetro da luz verdadeira e o da falsa luz pode influenciar na trombose da falsa luz.

**Figura Central:**
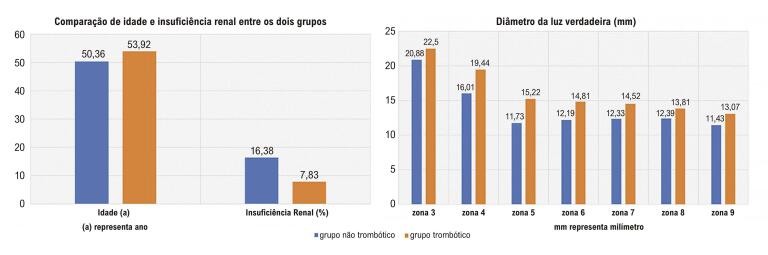
: Fatores que Afetam a Trombose da Falsa Luz na Dissecção Aórtica Tipo B

*Parâmetros com diferenças entre os dois grupos.*

## Introdução

A dissecção aórtica (DA) é um tipo de doença aórtica que se refere à separação da parede aórtica. Nesses casos, há uma ou mais rupturas na íntima aórtica, através das quais o sangue pode fluir para dentro ou para fora da falsa luz.^1^ A dissecção aórtica pode causar complicações graves envolvendo isquemia do órgão, como paraplegia e derrames, e pode até levar à ruptura aórtica. Os principais sintomas clínicos da dissecção aórtica são dor torácica, dor nas costas e síncope, que são facilmente confundidos com síndrome coronariana aguda, acidente vascular cerebral e embolia pulmonar e, consequentemente, a taxa de diagnóstico incorreto é de 33,8%.^2^

De acordo com a classificação de Stanford, a dissecção aórtica pode ser dividida em dissecção aórtica tipo A (DATA) e dissecção aórtica tipo B (DATB).^1^ Pacientes com dissecção aórtica tipo B representam 37,7% de todas as dissecções aórticas.^3^ A correção endovascular da aorta torácica (TEVAR) é um tratamento importante para DATB. A TEVAR envolve a cobertura da ruptura primária da dissecção aórtica por meio da implantação de uma endoprótese aórtica, reduzindo assim a pressão e a velocidade do fluido na falsa luz e induzindo trombose, levando ao remodelamento positivo da dissecção aórtica. A pesquisa mostrou que 91,3% dos pacientes com DATB submetidos a TEVAR apresentam remodelamento positivo.^4^ A trombose da falsa luz é um elo importante no remodelamento positivo da dissecção aórtica. A literatura também mostra que a trombose completa da falsa luz era um fator protetor para a dissecção aórtica^5^ e poderia beneficiar 90,6% dos pacientes.^6^ Isso mostra que a trombose completa da falsa luz é um excelente objetivo para a dissecção aórtica.

A relação entre a morfologia aórtica e o prognóstico permanece obscura. Kamman et al.^7^ sugeriram que um diâmetro aórtico reduzido está associado a um prognóstico ruim em pacientes com dissecção aórtica. No entanto, Spinelli et al.^5^ observaram uma associação entre um diâmetro máximo da aorta superior ao limiar de 40 a 41 mm e a dilatação da dissecção aórtica. Este estudo explora o papel das características morfológicas da dissecção aórtica na trombose da falsa luz em pacientes com DATB antes de serem submetidos ao tratamento com TEVAR.

## Método

### População do estudo

Trata-se de um estudo de coorte retrospectivo unicêntrico. Incluímos pacientes consecutivos diagnosticados com dissecção aórtica tipo B em nosso centro, no período entre janeiro de 2008 a dezembro de 2017. Todos os pacientes foram confirmados por exame de angiotomografia computadorizada (ATC). Os indivíduos foram divididos em um grupo trombótico e um grupo não trombótico, com base na detecção de qualquer trombo na falsa luz. Qualquer luz observada sem agente de contraste durante o exame de ATC foi considerada uma falsa luz trombosada, e qualquer luz na qual o meio de contraste e o trombo foram detectados foi considerada uma falsa luz parcialmente trombosada. Qualquer falsa luz que não contivesse um trombo foi considerada uma falsa luz patente. As falsas luzes trombosadas e parcialmente trombosadas foram incluídas no grupo trombótico, e as falsas luzes patentes foram incluídas no grupo não trombótico. Pacientes com dissecção aórtica tipo A, úlceras aórticas e hematoma intramural e pacientes com imagens de ATC não obtidas foram excluídos. Dados clínicos e informações demográficas de pacientes com dissecção aórtica tipo B foram obtidos a partir do sistema de prontuários do nosso hospital. Imagens de ATC de pacientes com dissecção aórtica tipo B estão disponíveis na estação de trabalho de imagem em nosso centro. Todas as imagens de ATC coletadas foram salvas em formato DICOM (sigla, em inglês, para Comunicação de Imagens Digitais em Medicina). Imagens da fase arterial foram utilizadas para nossas medições.

### Construção e medição de modelos tridimensionais

**Figura 1:**
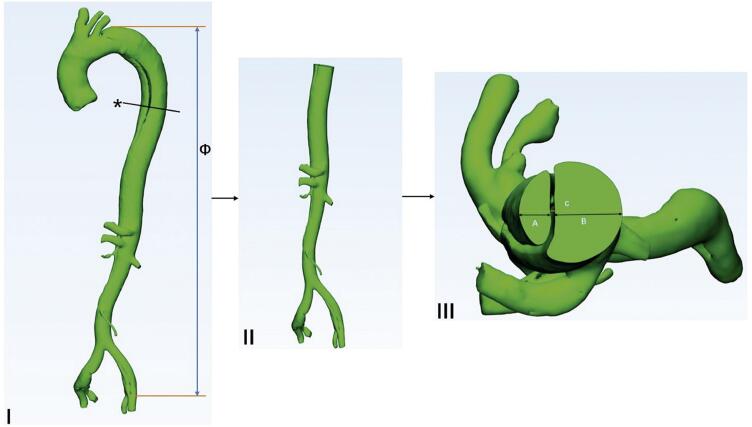
– Abordagem de medição aórtica. I) Modelo 3D de dissecação tipo B; II) Vista frontal; III) Vista superior. *: Corte transversal; ɸ: Comprimento vertical da dissecção aórtica; A: Diâmetro da luz verdadeira; B: Diâmetro da falsa luz; C: Diâmetro aórtico total.

O modelo tridimensional da dissecção aórtica foi reconstruído com o software MIMICS (versão 21.0, Materialise HQ, Leuven, Bélgica). As imagens de ATC foram importadas para o software MIMICS para segmentação e suavização. Por fim, um modelo tridimensional da dissecção aórtica foi produzido a partir dos três ramos do arco aórtico superior até o final da artéria ilíaca comum. Medimos o diâmetro máximo da luz verdadeira e falsa no início das zonas 0–11 de acordo com a divisão padrão da aorta.^1^ O comprimento vertical da dissecção aórtica foi medido e definido como a distância do ponto mais alto da dissecção até o ponto mais baixo, na direção vertical. As medições foram realizadas no modelo 3D. A abordagem de medição é mostrada na Figura 1.

### Análise estatística

Os dados categóricos são apresentados como valores absolutos e porcentagens. Dados contínuos são apresentados como média e desvio padrão (DP) ou mediana e intervalo interquartil, de acordo com a normalidade dos dados. O teste de Kolmogorov-Smirnov foi utilizado para avaliar a normalidade dos dados. O teste *t* de Student não pareado foi usado quando as variáveis contínuas eram normalmente distribuídas. Caso contrário, o teste U de Mann-Whitney foi usado. O teste do qui-quadrado foi utilizado para variáveis categóricas. Variáveis com p < 0,05 no teste *t* de Student não pareado, teste U de Mann-Whitney e teste do qui-quadrado foram incluídas na análise de regressão logística binária para análise multivariada. Valores de p < 0,05 foram considerados estatisticamente significativos. As análises estatísticas foram realizadas no software SPSS (versão 25.0).

## Resultados

**Figura 2:**
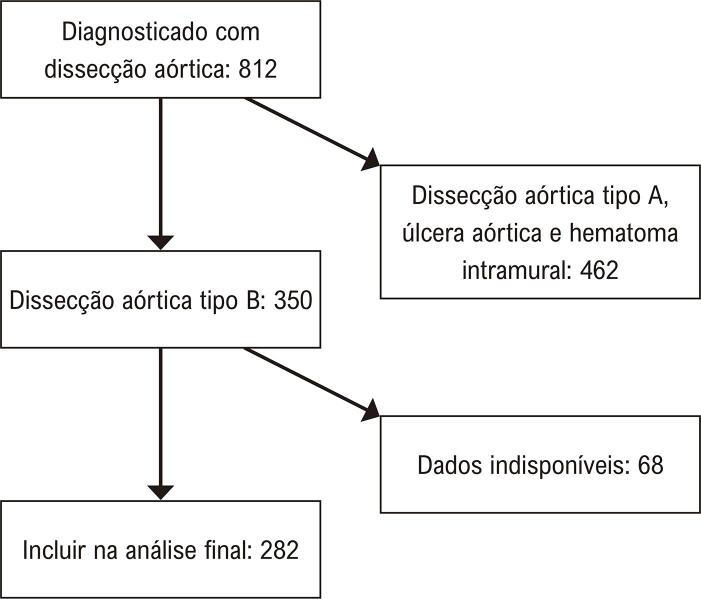
– *Processo de triagem dos participantes.*

No período selecionado de dez anos, 812 pacientes foram identificados com dissecção aórtica, 350 dos quais foram diagnosticados com dissecção aórtica tipo B. Informações clínicas e de imagem não estavam disponíveis para 68 pacientes, resultando em 282 pacientes que preencheram nossos critérios de inclusão. O processo de triagem é resumido na Figura 2. A média de idade dos pacientes foi de 52,45 ± 11,24, sendo 83,69% dos pacientes do sexo masculino. Os pacientes do grupo trombótico eram mais velhos do que os do grupo não trombótico, 53,92 ± 11,40 vs. 50,36 ± 10,71, respectivamente (p < 0,05). A insuficiência renal foi mais comum no grupo não trombótico do que no grupo trombótico (p < 0,05). As características demográficas e clínicas dos casos incluídos são apresentadas na Tabela 1.

**Tabela 1:** – Características clínicas e demográficas do paciente

Características	Grupo não trombótico (n=116)(%)	Grupo trombótico (n=166)(%)	Valor de p
Masculino	98 (84,48)	138 (83,13)	0,763
Idade (anos)	50,36±10,71	53,92±11,40	0,009
Hipertensão	106 (91,38)	150 (90,36)	0,771
Tabagismo	42 (36,21)	61 (36,75)	0,926
Diabetes melito	6 (5,17)	17 (10,24)	0,126
Acidente vascular encefálico	11 (9,48)	19 (11,45)	0,599
Insuficiência Renal	19 (16,38)	13 (7,83)	0,026
Doença arterial coronariana	6 (5,17)	20 (12,05)	0,050
PAS no momento da admissão (mmhg)	151,44±29,59	152,02±27,21	0,864
PAD no momento da admissão (mmhg)	89,51±20,91	87,56±16,56	0,404
Tempo do início dos sintomas até a ATC (h)	95 (27,0-336,0)	96 (24,0-240,0)	0,184
IMC (kg/m^2^)	25,15±5,20	24,59 (22,34-27,41)	0,998

*PAS: pressão arterial sistólica; PAD: pressão arterial diastólica; ATC: *angiotomografia computadorizada*; IMC: índice de massa corporal.*

Os resultados morfológicos são apresentados na Tabela 2. A taxa de envolvimento retrógrado do arco aórtico foi de 7,45%, com 4,96% atingindo a zona 1 e 1,77% atingindo a zona 0. Na zona 0, o diâmetro da luz verdadeira no grupo não trombótico foi significativamente maior que no grupo trombótico. Nas zonas 3-9, o diâmetro da luz verdadeira foi menor do que no grupo trombótico. Entretanto, o diâmetro da falsa luz foi maior no grupo não trombótico do que no grupo trombótico nas zonas 4 e 5. A figura central mostra os parâmetros que diferiram entre os dois grupos. De acordo com os resultados da análise de regressão logística binária, o diâmetro da luz verdadeira na zona 5 e a insuficiência renal foram preditores independentes de trombose da falsa luz. Os resultados são apresentados na Tabela 3.

**Tabela 2:** – Características morfológicas

Variáveis (mm)		Grupo não trombótico (n=116)	Grupo trombótico (n=166)	Valor de p
Comprimento vertical da dissecção aórtica		351,80±101,62	345,38±92,56	0,582
Zona 0	Diâmetro da luz verdadeira	32,44±5,62	30,80±5,24	0,014
	Diâmetro da falsa luz	0 (0)	0 (0)	**0,007**
	Diâmetro aórtico total	32,44±5,62	31,65±5,12	0,233
Zona 1	Diâmetro da luz verdadeira	30,46±3,42	29,27±4,55	0,130
	Diâmetro da falsa luz	0 (0)	0 (0-0)	0,113
	Diâmetro aórtico total	30,71±3,42	29,96±4,24	0,103
Zona 2	Diâmetro da luz verdadeira	28,34±3,16	27,59±4,49	0,123
	Diâmetro da falsa luz	0 (0)	0 (0)	0,081
	Diâmetro aórtico total	28,82±3,68	28,56±4,13	0,585
Zona 3	Diâmetro da luz verdadeira	20,88±6,16	22,50±5,79	**0,026**
	Diâmetro da falsa luz	9,74±8,22	8,71±6,55	0,244
	Diâmetro aórtico total	31,65±6,78	31,80±5,83	0,841
Zona 4	Diâmetro da luz verdadeira	16,01±7,62	19,44±7,91	**0,000**
	Diâmetro da falsa luz	19,97±11,31	16,37±10,31	**0,007**
	Diâmetro aórtico total	37,57±8,73	36,91±7,93	0,513
Zona 5	Diâmetro da luz verdadeira	11,73±4,83	15,22±6,44	**0,000**
	Diâmetro da falsa luz	19,34±7,76	16,71±10,14	**0,019**
	Diâmetro aórtico total	32,88±6,08	32,98±7,81	0,907
Zona 6	Diâmetro da luz verdadeira	12,19±4,56	14,81±5,56	**0,000**
	Diâmetro da falsa luz	15,39±6,62	13,89±8,19	0,472
	Diâmetro aórtico total	29,53±4,74	29,89±6,01	0,598
Zona 7	Diâmetro da luz verdadeira	12,33±5,15	14,52±5,50	**0,001**
	Diâmetro da falsa luz	13,91±7,19	12,20±7,63	**0,060**
	Diâmetro aórtico total	27,87±4,75	27,76±5,40	0,866
Zona 8	Diâmetro da luz verdadeira	12,39±5,11	13,81±5,11	**0,022**
	Diâmetro da falsa luz	11,68±6,98	10,14±7,36	0,077
	Diâmetro aórtico total	25,39±5,25	25,16±5,60	0,724
Zona 9	Diâmetro da luz verdadeira	11,43±4,78	13,07±4,67	**0,004**
	Diâmetro da falsa luz	10,28±7,00	8,60±7,38	0,056
	Diâmetro aórtico total	23,23±4,89	22,69±5,30	0,386
Zona 10	Diâmetro da luz verdadeira	12,11±5,26	12,98±5,10	0,169
	Diâmetro da falsa luz	8,18±7,93	5,44 (0-11,06)	0,378
	Diâmetro aórtico total	21,53±6,49	21,01±8,27	0,560
Zona 11 (esquerda)	Diâmetro da luz verdadeira	10,10±3,62	9,93±3,17	0,685
	Diâmetro da falsa luz	0 (0-4,05)	0 (0-2,03)	0,712
	Diâmetro aórtico total	12,86±3,72	12,51±4,07	0,446
Zona 11 (direita)	Diâmetro da luz verdadeira	10,29±3,90	10,43±3,22	0,748
	Diâmetro da falsa luz	0 (0-5,15)	0 (0)	0,335
	Diâmetro aórtico total	13,61±4,48	12,93±4,50	0,218

**Tabela 3:** – Análise de regressão logística binária

Variáveis	B	Wald	Valor de p	OR (IC de 95%)
Idade	0,008	0,370	0,543	1,008 (0,982-1,035)
Doença arterial coronariana	-0,792	2,114	0,146	0,453 (0,156-1,317)
Insuficiência renal	0,874	4,242	0,039	2,397 (1,043-5,508)
Diâmetro da luz verdadeira na zona 0	-0,049	2,672	0,102	0,952 (0,898-1,010)
Diâmetro da falsa luz na zona 0	2,580	0,000	0,998	13,202 (0,000)
Diâmetro da luz verdadeira na zona 3	-0,010	0,110	0,740	0,990 (0,935-1,049)
Diâmetro da luz verdadeira na zona 4	0,017	0,336	0,562	1,017(0,960-1,078)
Diâmetro da falsa luz na zona 4	-0,006	0,098	0,754	0,994 (0,957-1,033)
Diâmetro da luz verdadeira na zona 5	0,102	6,676	0,010	1,108 (1,025-1,197)
Diâmetro da falsa luz na zona 5	0,025	1,371	0,242	1,025 (0,983-1,069)
Diâmetro da luz verdadeira na zona 6	0,022	0,218	0,640	1,023 (0,931-1,124)
Diâmetro da luz verdadeira na zona 7	0,022	0,191	0,662	1,023 (0,925-1,131)
Diâmetro da luz verdadeira na zona 8	-0,063	1,360	0,243	0,939 (0,845-1,044)
Diâmetro da luz verdadeira na zona 9	0,051	1,080	0,299	1,052 (0,956-1,157)

*OR: odds ratio; IC: intervalo de confiança.*

**Figura 3:**
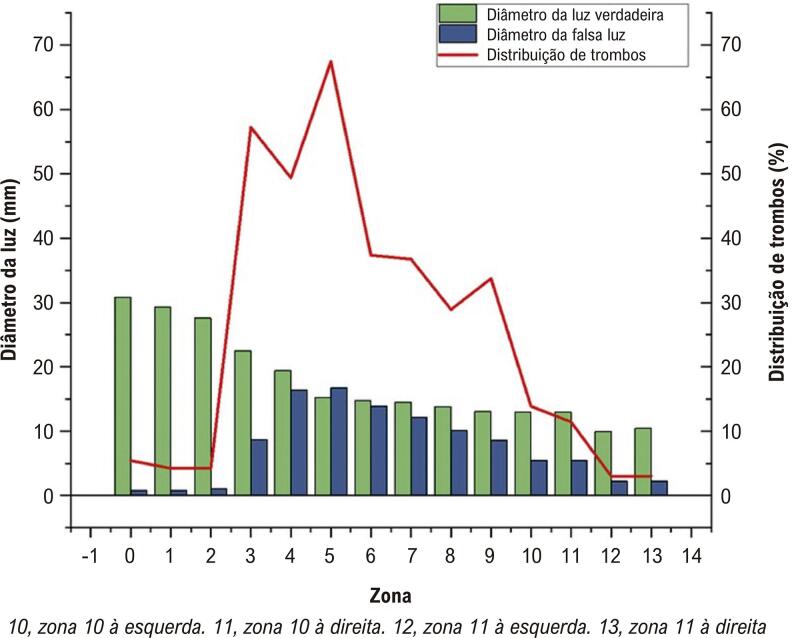
– *Diâmetros da luz verdadeira e falsa e distribuição do trombo nas zonas 0-11.*

No grupo trombótico, também focamos na distribuição do trombo em cada zona. A zona 5 foi o local mais comum do trombo (67,47%), seguida da zona 3 (57,23%), e a zona 11 foi a área com menor distribuição de trombo, com apenas 3,01%. A relação correspondente entre a distribuição do trombo e o diâmetro da luz verdadeira e falsa é mostrada na Figura 3.

## Discussão

A dissecção aórtica tipo B é definida como a ruptura de entrada primária com origem na região esquerda do tronco braquiocefálico, incluindo o arco aórtico e a aorta descendente.^1^ A incidência foi de 1,6 a cada 100.000 pessoas por ano.^8^ A DATB pode causar síndrome de má perfusão e ruptura aórtica, que pode ser fatal. A mortalidade intra-hospitalar devido à dissecção aórtica tipo B é de 0,3 por 100.000 por ano.^8^ A DA aumenta a taxa de mortalidade cardiovascular. A mortalidade cardiovascular em pacientes com DA é 2-3 vezes maior do que na população em geral.^9^

Durante os dez anos de nosso estudo, 282 pacientes preencheram nossos critérios de inclusão, dos quais 83,69% eram do sexo masculino. Esse número foi maior do que os 69,1% relatados na literatura.^3^ A razão pode ser o maior número de fumantes de tabaco na China do que em outros países. A OMS relatou que a porcentagem de fumantes na China é de 47,6%, em comparação com 19% nos Estados Unidos.^10^ Na China, descobriu-se que o número de homens que fumam é 22 vezes maior do que o número de mulheres que fumam.^11^ Descobriu-se que fumar é um fator de alto risco para dissecção aórtica. O mecanismo subjacente a essa conexão é que o extrato da fumaça de cigarro causa a morte das células da musculatura lisa vascular por induzir a ferroptose.^12^

O termo “insuficiência renal” aqui se refere a uma taxa de filtração glomerular estimada [eGFR] inferior a 89 mL/min/1,73 m^2^. A prevalência de insuficiência renal foi significativamente menor no grupo trombótico do que no grupo não trombótico (7,83% vs. 16,38%, p < 0,05). Essa diferença pode ocorrer porque a insuficiência renal pode causar um declínio na eficiência do sistema circulatório, e pacientes com doença renal em estágio terminal apresentam contagens de leucócitos e plaquetas mais baixas do que pacientes sem doença renal em estágio terminal.^13^ Sakakura et al.^14^ também descobriram que a insuficiência renal é um preditor de resultados adversos a longo prazo em pacientes com DATB. No entanto, pacientes com DATB com insuficiência renal exibiram mais sintomas atípicos do que os pacientes com DATB.^13^ Recomenda-se aos médicos prestar mais atenção aos pacientes com insuficiência renal e dissecção aórtica.

Para avaliar a relação entre o diâmetro da aorta e a trombose da falsa luz, medimos o diâmetro da luz verdadeira, o diâmetro da falsa luz e o diâmetro total da aorta no início de cada zona. Nas zonas 3–9, o diâmetro da luz verdadeira do grupo trombótico foi maior em comparação com o grupo não trombótico. Além disso, o diâmetro da luz verdadeira é maior que o diâmetro da falsa luz, sendo mais comum no grupo trombótico. Especulamos que, quando o diâmetro da luz verdadeira é maior que o diâmetro da falsa luz, as condições favorecem a trombose da falsa luz. No entanto, quando o diâmetro da luz verdadeira é menor que o da falsa luz, seria mais provável a falsa luz estar em estado de patência. Este resultado coincide com pesquisas anteriores. Matsushita et al.^15^ mostraram que era mais comum a falsa luz ser maior do que a luz verdadeira em pacientes com falsa luz patente e que isso poderia ser um preditor de eventos adversos aórticos importantes. Os principais eventos adversos aórticos incluem morte relacionada à aorta, cirurgia de dissecção aórtica tardia e aumento rápido da falsa luz. Muitos fatores têm sido relacionados ao estado da falsa luz, e o monitoramento regular pode ter efeitos benéficos no prognóstico.^16^

As diretrizes atuais recomendam terapia médica ideal (OMT) para o controle da frequência cardíaca e hipertensão como tratamento de primeira linha para dissecção aórtica tipo B aguda não complicada sem evidência de ruptura ou má perfusão do órgão.^17^ No entanto, durante o acompanhamento, mais de 70% dos pacientes tratados com OMT apresentaram remodelamento negativo,^3^ e 26,2% dos pacientes necessitaram de reintervenção.^18^ Os médicos têm favorecido a TEVAR porque ela supera a OMT em termos de melhoria da remodelação da dissecção aórtica.^4^ O principal mecanismo subjacente ao remodelamento positivo induzido pela TEVAR em lesões dissecantes é a indução de alterações na hemodinâmica das luzes verdadeira e falsa. Após a implantação da endoprótese, enquanto dilata mecanicamente a luz verdadeira, a falsa luz é comprimida, o que pode melhorar a perfusão sanguínea e aumentar a pressão na luz verdadeira. No entanto, uma vez que a ruptura primária fosse coberta, o fluxo sanguíneo e a velocidade na falsa luz seriam reduzidos, resultando em alterações nas características do fluxo sanguíneo na falsa luz e, assim, contribuindo para a formação de trombos.^19^ No entanto, existem limitações quanto ao uso da TEVAR para dissecção envolvendo ramos importantes e, portanto, novas técnicas e dispositivos precisam ser desenvolvidos.

### Limitações

O presente trabalho apresentou várias limitações. Trata-se de um estudo retrospectivo de centro único, com tamanho amostral limitado; estudos prospectivos de longo prazo e amostragem grande revelariam melhor o prognóstico da dissecção aórtica tipo B. Diversos fatores morfológicos afetam o estado da falsa luz e, portanto, mais características morfológicas devem ser analisadas. Além disso, as medições foram realizadas com base em imagens de ATC e, portanto, a qualidade de tais imagens pode afetar a precisão das medições.

## Conclusões

Neste estudo, descobrimos que a incidência da falsa luz trombótica foi maior em pacientes idosos com função renal normal em comparação com pacientes mais jovens ou com função renal comprometida. O diâmetro da luz verdadeira da aorta descendente foi relacionado à trombose na falsa luz.
