# Supplementary material for: Fatores que Afetam a Trombose da Falsa Luz na Dissecção Aórtica Tipo B
Source: Arq Bras Cardiol. 2023 Jul 20;120(8):e20220939. [Article in Portuguese] doi: 10.36660/abc.20220939 (PMC10374264; doi:10.36660/abc.20220939)
Supplement: Translation

# Translation: Factors Affecting False Lumen Thrombosis In Type B Aortic Dissection

**Authors:** Qian-hui Tang, Jing Chen, Han Yang, Zhong Qin, Qiu-ning Lin, Xiao Qin

**Affiliations:** 1 China Department of Vascular and Endovascular Surgery – The First Affiliated Hospital of Guangxi Medical University, Guangxi – China

**Keywords:** Aortic Dissection, Thrombosis, Light

## Abstract

**Background:**

Complete thrombosis of the false lumen facilitates remodeling of type B aortic dissection (TBAD). Morphological characteristics affect thrombosis in the false lumen.

**Objectives:**

Discuss the factors present before admission that influence false lumen thrombosis in patients with TBAD.

**Methods:**

We studied 282 patients diagnosed with TBAD in our hospital between January 2008 and December 2017. We divided the subjects into a thrombotic group and a non-thrombotic group based on whether any thrombus was detectable in the false lumen. We analyzed the differences between the two groups with respect to clinical data, the vertical length of the dissection, and the diameter of the aorta. P values < 0.05 were considered statistically significantly different.

**Results:**

Significant differences between the thrombotic group and non-thrombotic group were found with respect to age (53.92 ± 11.40 vs. 50.36 ± 10.71, p = 0.009) and proportion of patients with renal insufficiency (7.83% vs. 16.38%, p = 0.026). In zones 3–9, the true lumen diameter of the thrombotic group was significantly larger than in the non-thrombotic group (p < 0.05). Binary logistic regression analysis showed that true lumen diameter in zone 5 and renal insufficiency were independent predictors of false lumen thrombosis.

**Conclusions:**

Age and renal function were associated with thrombosis in the false lumen. Potentially, the difference between the diameter of the true lumen diameter and that of the false lumen may influence the thrombosis of the false lumen.

**Central Illustration:** : Factors Affecting False Lumen Thrombosis In Type B Aortic Dissection Parameters with differences between the two groups.

## Introduction

Aortic dissection (AD) is a type of aortic disease that refers to the separation of the aortic wall. In such cases, there are one or more tears in the aortic intima through which blood can flow into or out of the false lumen.^1^ Aortic dissection can cause serious complications involving organ ischemia, such as paraplegia and strokes, and it can even lead to aortic rupture. As the main clinical symptoms of aortic dissection are chest pain, back pain, and syncope, which are easily confused with acute coronary syndrome, stroke, and pulmonary embolism, the misdiagnosis rate is 33.8%.^2^

According to Stanford classification, aortic dissection can be divided into type A aortic dissection (TAAD) and type B aortic dissection (TBAD).^1^ Patients with type B aortic dissection account for 37.7% of all aortic dissections.^3^ Thoracic endovascular aortic repair (TEVAR) is an important treatment for TBAD. TEVAR involves covering the primary tear of the aortic dissection by implanting a stent-graft in the aorta, thereby reducing the pressure and velocity of the fluid in the false lumen and inducing thrombosis, leading to positive remodeling of the aortic dissection. Research has shown that 91.3% of TBAD patients who undergo TEVAR experience positive remodeling.^4^ False lumen thrombosis is an important link in the positive remodeling of aortic dissection. Previous literature has also shown that complete false lumen thrombosis was a protective factor for aortic dissection^5^ and could benefit 90.6% of patients.^6^ This shows that complete false lumen thrombosis is an excellent goal for aortic dissection.

The relationship between aortic morphology and prognosis remains unclear. Kamman et al.^7^ suggested that a small aortic diameter is associated with poor prognosis in patients with aortic dissection. However, Spinelli et al.^5^ found a maximum diameter of the aorta exceeding the threshold of 40 to 41 mm to be associated with dilation of aortic dissection. This study explores the role of morphological characteristics of aortic dissection in the thrombosis of the false lumen in TBAD patients before they undergo TEVAR treatment.

## Methods

### Study population

This is a single-center retrospective cohort study. We included consecutive patients diagnosed with type B aortic dissection in our center from January 2008 to December 2017. All patients were confirmed by computed tomographic angiography (CTA) examination. We divided them into a thrombotic group and a non-thrombotic group according to whether any thrombus was found in the false lumen. Any lumen found containing no contrast agent during CTA examination was considered a thrombosed false lumen, and any lumen in which both contrast medium and thrombus were detected was considered a partially thrombosed false lumen. Any false lumen that did not contain a thrombus was considered a patent false lumen. The thrombosed and partially thrombosed false lumens were placed in the thrombotic group, and patent false lumens were placed in the non-thrombotic group. Patients with type A aortic dissection, aortic ulcers, and intramural hematoma and patients with unobtainable CTA images were excluded. Clinical data and demographic information of patients with type B aortic dissection were obtained from our hospital medical record system. CTA images of patients with type B aortic dissection are available on our center’s imaging workstation. All collected CTA images were saved in digital imaging and communications in medicine (DICOM) format. Images of the arterial phase were used for our measurements.

### Three-dimensional model construction and measurement

The three-dimensional model of aortic dissection was reconstructed with the MIMICS (version 21.0, Materialise HQ, Leuven, Belgium) software. The CTA images were imported into the MIMICS software for segmentation and smoothing. Finally, a three-dimensional model of aortic dissection from the three branches of the superior aortic arch to the end of the common iliac artery was produced. We measured the maximum diameter of the true and false lumen at the beginning of zones 0–11 according to the standard division of the aorta.^1^ The vertical length of the aortic dissection was measured and defined as the distance from the highest point of the aortic dissection to the lowest point in the vertical direction. The measurements were performed on the 3D model. The measurement approach is shown in Figure 1.

**Figure 1:** – Aortic measurement approach. I) Type B dissection 3D model; II) Front view; III) Top view. *: Cross section; ɸ: aortic dissection vertical length; A: True lumen diameter; B: False lumen diameter; C: Total aortic diameter.

### Statistical analysis

Categorical data is presented as absolute values and percentages. Continuous data is presented as mean and standard deviation (SD) or median and interquartile range according to data normality. The Kolmogorov-Smirnov test was used to assess the normality of the data. The unpaired student’s *t* test was used when continuous variables were normally distributed. Otherwise, the Mann–Whitney U test was used. The chi-square test was used for categorical variables. Variables with p <0.05 in unpaired Student’s t test, Mann–Whitney U test, and chi-square test were included in binary logistic regression analysis for multivariate analysis. P values < 0.05 were considered statistically significant. Statistical analyses were conducted using SPSS software (version 25.0).

## Results

In the chosen ten-year period, 812 patients were considered to suffer from aortic dissection, 350 of whom were diagnosed with type B aortic dissection. Clinical and imaging information was unavailable for 68 patients, which left 282 patients who met our inclusion criteria. The screening process is summarized in Figure 2. The mean age of the patients was 52.45 ± 11.24, with 83.69% of patients being male. The patients in the thrombotic group were older than those in the non-thrombotic group, 53.92 ± 11.40 vs. 50.36 ± 10.71, respectively (p < 0.05). Renal insufficiency was more common in the non-thrombotic group than in the thrombotic group (p < 0.05). The demographics and clinical characteristics of the included cases are presented in Table 1.

**Figure 2:** – *The screening process of participants.*

**Table 1:** – Patient clinical and demographic characteristics

Characteristic	non-thrombotic group (n=116)(%)	thrombus group (n=166)(%)	p value
Male	98 (84.48)	138 (83.13)	0.763
Age (years)	50.36±10.71	53.92±11.40	0.009
Hypertension	106 (91.38)	150 (90.36)	0.771
Smoking	42 (36.21)	61 (36.75)	0.926
Diabetes mellitus	6 (5.17)	17 (10.24)	0.126
Stroke	11 (9.48)	19 (11.45)	0.599
Renal insufficiency	19 (16.38)	13 (7.83)	0.026
Coronary artery disease	6 (5.17)	20 (12.05)	0.050
SBP on admission (mmhg)	151.44±29.59	152.02±27.21	0.864
DBP on admission (mmhg)	89.51±20.91	87.56±16.56	0.404
Time from onset to CTA examination (h)	95 (27.0-336.0)	96 (24.0-240.0)	0.184
BMI (kg/m^2^)	25.15±5.20	24.59 (22.34-27.41)	0.998

*SBP: systolic blood pressure; DBP: diastolic blood pressure; CTA: *computed tomographic angiography*; BMI: body mass index.*

Morphological results are presented in Table 2. The rate of retrograde involvement of the aortic arch was 7.45%, with 4.96% reaching zone 1 and 1.77% reaching zone 0. In zone 0, the diameter of the true lumen in the non-thrombotic group was significantly larger than in the thrombotic group. In zones 3–9, the true lumen diameter was smaller than that in the thrombotic group. However, the diameter of the false lumen was larger in the non-thrombotic group than thrombotic group in zones 4 and 5. The central figure shows the parameters that differed between the two groups. According to binary logistic regression analysis results, true lumen diameter in zone 5 and renal insufficiency were independent predictors of false lumen thrombosis. Results are presented in Table 3.

**Table 2:** – Morphological characteristics

Variables (mm)		Non-thrombotic group (n=116)	Thrombus group (n=166)	p value
Vertical length of aortic dissection		351.80±101.62	345.38±92.56	0.582
Zone 0	True lumen diameter	32.44±5.62	30.80±5.24	0.014
	False lumen diameter	0 (0)	0 (0)	**0.007**
	Total aortic diameter	32.44±5.62	31.65±5.12	0.233
Zone 1	True lumen diameter	30.46±3.42	29.27±4.55	0.130
	False lumen diameter	0 (0)	0 (0-0)	0.113
	Total aortic diameter	30.71±3.42	29.96±4.24	0.103
Zone 2	True lumen diameter	28.34±3.16	27.59±4.49	0.123
	False lumen diameter	0 (0)	0 (0)	0.081
	Total aortic diameter	28.82±3.68	28.56±4.13	0.585
Zone 3	True lumen diameter	20.88±6.16	22.50±5.79	**0.026**
	False lumen diameter	9.74±8.22	8.71±6.55	0.244
	Total aortic diameter	31.65±6.78	31.80±5.83	0.841
Zone 4	True lumen diameter	16.01±7.62	19.44±7.91	**0.000**
	False lumen diameter	19.97±11.31	16.37±10.31	**0.007**
	Total aortic diameter	37.57±8.73	36.91±7.93	0.513
Zone 5	True lumen diameter	11.73±4.83	15.22±6.44	**0.000**
	False lumen diameter	19.34±7.76	16.71±10.14	**0.019**
	Total aortic diameter	32.88±6.08	32.98±7.81	0.907
Zone 6	True lumen diameter	12.19±4.56	14.81±5.56	**0.000**
	False lumen diameter	15.39±6.62	13.89±8.19	0.472
	Total aortic diameter	29.53±4.74	29.89±6.01	0.598
Zone 7	True lumen diameter	12.33±5.15	14.52±5.50	**0.001**
	False lumen diameter	13.91±7.19	12.20±7.63	**0.060**
	Total aortic diameter	27.87±4.75	27.76±5.40	0.866
Zone 8	True lumen diameter	12.39±5.11	13.81±5.11	**0.022**
	False lumen diameter	11.68±6.98	10.14±7.36	0.077
	Total aortic diameter	25.39±5.25	25.16±5.60	0.724
Zone 9	True lumen diameter	11.43±4.78	13.07±4.67	**0.004**
	False lumen diameter	10.28±7.00	8.60±7.38	0.056
	Total aortic diameter	23.23±4.89	22.69±5.30	0.386
Zone 10	True lumen diameter	12.11±5.26	12.98±5.10	0.169
	False lumen diameter	8.18±7.93	5.44 (0-11.06)	0.378
	Total aortic diameter	21.53±6.49	21.01±8.27	0.560
Zone 11 (left)	True lumen diameter	10.10±3.62	9.93±3.17	0.685
	False lumen diameter	0 (0-4.05)	0 (0-2.03)	0.712
	Total aortic diameter	12.86±3.72	12.51±4.07	0.446
Zone 11 (right)	True lumen diameter	10.29±3.90	10.43±3.22	0.748
	False lumen diameter	0 (0-5.15)	0 (0)	0.335
	Total aortic diameter	13.61±4.48	12.93±4.50	0.218

*OR: odds ratio; CI: confidence interval.*

**Table 3:** – Binary logistic regression analysis

Variables	B	Wald	p value	OR(95% CI)
Age	0.008	0.370	0.543	1.008 (0.982-1.035)
Coronary artery disease	-0.792	2.114	0.146	0.453 (0.156-1.317)
Renal insufficiency	0.874	4.242	0.039	2.397 (1.043-5.508)
True lumen diameter in zone 0	-0.049	2.672	0.102	0.952 (0.898-1.010)
False lumen diameter in zone 0	2.580	0.000	0.998	13.202 (0.000)
True lumen diameter in zone 3	-0.010	0.110	0.740	0.990 (0.935-1.049)
True lumen diameter in zone 4	0.017	0.336	0.562	1.017(0.960-1.078)
False lumen diameter in zone 4	-0.006	0.098	0.754	0.994 (0.957-1.033)
True lumen diameter in zone 5	0.102	6.676	0.010	1.108 (1.025-1.197)
False lumen diameter in zone 5	0.025	1.371	0.242	1.025 (0.983-1.069)
True lumen diameter in zone 6	0.022	0.218	0.640	1.023 (0.931-1.124)
True lumen diameter in zone 7	0.022	0.191	0.662	1.023 (0.925-1.131)
True lumen diameter in zone 8	-0.063	1.360	0.243	0.939 (0.845-1.044)
True lumen diameter in zone 9	0.051	1.080	0.299	1.052 (0.956-1.157)

In the thrombotic group, we also focused on the distribution of thrombus in each zone. Zone 5 was the most common site of thrombus (67.47%), followed by zone 3 (57.23%), and zone 11 was the area with the least thrombus distribution, only 3.01%. The corresponding relationship between the distribution of thrombus and the diameter of the true and false lumen diameter of the zone is shown in Figure 3.

**Figure 3:** – *True and false lumen diameters and thrombus distribution in zones 0-11.*

## Discussion

Type B aortic dissection is defined as the primary entry tear originating from the left region of the innominate artery, including the aortic arch and descending aorta.^1^ The incidence was 1.6 per 100,000 per year.^8^ TBAD can cause mal-perfusion syndrome and aortic rupture, which can be life-threatening. The in-hospital mortality due to type B aortic dissection has been reported to be 0.3 per 100 000 per year.^8^ AD increases cardiovascular mortality. It has been reported that cardiovascular mortality in patients with AD is 2-3 times higher than in the general population.^9^

During the ten years of our study, 282 patients met our inclusion criteria, of whom 83.69% were male. This was higher than the 69.1% reported in the literature.^3^ This may be because there are more tobacco smokers in China than in other countries. The WHO has reported the percentage of smokers in China to be 47.6%, compared to 19% in the United States.^10^ In China, the number of men who smoke has been found to be 22 times higher than the number of women who smoke.^11^ Smoking has been found to be a high-risk factor for aortic dissection. The mechanism underlying this connection is that cigarette smoke extract causes vascular smooth muscle cell death by inducing ferroptosis.^12^

The term “renal insufficiency” here refers to an estimated glomerular filtration rate [eGFR] under 89 mL/min/1.73 m^2^. The prevalence of renal insufficiency was significantly lower in the thrombotic group than in the non-thrombotic group (7.83% vs. 16.38%, p < 0.05). This difference may occur because renal insufficiency can cause a decline in the efficiency of the circulatory system, and patients with end-stage renal disease were found to have lower white blood cell and platelet counts than patients without end-stage renal disease.^13^ Sakakura et al.^14^ also found that renal insufficiency is a predictor of long-term adverse outcomes in patients with TBAD. However, TBAD patients with renal insufficiency exhibited more atypical symptoms than their counterparts with TBAD.^13^ Physicians should pay more attention to patients with both renal insufficiency and aortic dissection.

To assess the relationship between aortic diameter and false lumen thrombosis, we measured true lumen diameter, false lumen diameter, and total aortic diameter at the beginning of each zone. In zones 3–9, the true lumen diameter of the thrombotic group was larger than that of the non-thrombotic group. In addition, the true lumen diameter is larger than the false lumen diameter, which is more common in the thrombotic group. We speculated that when the diameter of the true lumen was larger than the diameter of the false lumen, conditions would favor thrombosis in the false lumen. However, when the diameter of the true lumen was smaller than that of the false lumen, the false lumen would be more likely to be in a patency state. This result coincides with previous research. Matsushita et al.^15^ showed that it was more common for the false lumen to be larger than the true lumen in patients with a patent false lumen and that it could be a predictor of major aortic-related adverse events. Major aortic-related adverse events include aortic-related death, late aortic dissection surgery, and rapid false lumen enlargement. Many factors have been found to be related to the state of the false lumen, and regular monitoring may have beneficial effects on prognosis.^16^

Current guidelines recommend optimal medical therapy (OMT) for heart rate and hypertension control as a first-line treatment for acute uncomplicated type B aortic dissection without evidence of rupture or organ mal-perfusion.^17^ However, during follow-up, more than 70% of patients treated with OMT experienced negative remodeling,^3^ and 26.2% of patients required reintervention.^18^ Clinicians favor TEVAR because it outperforms OMT in improving the remodeling of aortic dissection.^4^ The main mechanism underlying TEVAR-induced positive remodeling in dissecting lesions is the induction of changes in the hemodynamics of true and false lumens. After stent-graft implantation, while mechanically dilating the true lumen, the false lumen is compressed, which can improve blood perfusion and increase the pressure in the true lumen. However, once the primary tear was covered, the blood flow and velocity into the false lumen would be reduced, resulting in changes to blood flow characteristics in the false lumen and therefore contributing to the formation of thrombus in the false lumen.^19^ However, there are limitations to the use of TEVAR for dissection involving important branch vessels, so newer techniques and devices need to be developed.

### Limitations

The present paper has several limitations. This is a single-center retrospective study with limited sample size; long-term, large-sample prospective studies would better reveal the prognosis of type B aortic dissection. Many morphological factors affect the state of the false lumen, so more morphological features should be analyzed. We also took measurements based on CTA images, so the quality of the CTA images may affect the accuracy of the measurements.

## Conclusions

In this study, we found that the incidence of thrombotic false lumen was higher in older patients with normal renal function than in patients who were younger or had compromised kidney function. The diameter of the true lumen of the descending aorta was related to the thrombosis in the false lumen.
